# Development and assessment of a curriculum model for virtual simulation in nursing: curriculum development and pilot-evaluation

**DOI:** 10.1186/s12909-023-04283-4

**Published:** 2023-04-26

**Authors:** Hyunsook Shin, Dahae Rim

**Affiliations:** College of Nursing Science, Kyung Hee University, Seoul, South Korea

**Keywords:** Nursing education, Simulation, Curriculum, Virtual simulation, Simulation

## Abstract

**Background:**

To introduce virtual simulation as a strategy of nursing education and provide valid educational content, the best curriculum model of virtual simulation needs to be developed.

**Methods:**

Curriculum development process and pilot evaluation was used. The curriculum content and structure was developed by analyzing literature including previous studies and major nursing classification systems, and key words derived from focus group interviews of 14 nurses and 20 faculty members with expertise in simulation education. 35 nursing students participated in the evaluation of the developed virtual simulation curriculum.

**Results:**

The curriculum developed for virtual simulation in nursing education contained three domains of content areas: (1) enhancing clinical decision-making, (2) experiencing low-exposed situations, and (3) building professional resilience. In addition, seven subdomains of content areas and 35 representative topics in the virtual simulation curriculum were derived. Scenarios of nine representative topics were created, translated into 3D modeling and pilot-evaluated.

**Conclusions:**

Considering that nursing education is encountering new demands and challenges from students and the changing society, the newly suggested curriculum for virtual nursing simulation can help nurse educators to plan better educational opportunities for students.

**Supplementary Information:**

The online version contains supplementary material available at 10.1186/s12909-023-04283-4.

## Background

The current healthcare system demands for professionals with metacognitive ability and creative talents. To meet these demands, nursing education has continuously pursued changes in the academic environment by developing and applying innovative teaching methods [1, 2]. Metacognitive abilities were defined as learning management ability to understand and manipulate one’s cognitive activities[3]. Considering that nursing education is mostly focused on systematic problem-solving using metacognitive abilities, present educational techniques (e.g., classroom lectures and practicum experience in clinical spaces) where observation is the major learning method for nursing students are increasingly restricted in terms of enhancing their metacognition [4, 5]. To overcome these problems, various teaching and learning strategies such as simulation have been developed and operationalized[6]. However, recently demand for virtual simulation has been increased due to space and time constraints, inadequate human resources, and environmental constraints for realistic learning experiences. In addition, virtual simulation can act as one of alternative strategies in situations where learners’ access to clinical practice was restricted such as the current Pandemic. In order to apply virtual simulation properly to improve metacognitive ability, the development of effective pedagogies is required .

Virtual simulation has been used as an umbrella term to describe virtual modalities but in a recent systematic review[7], it is defined as partially immersive, screen-based experiences whereas virtual reality or virtual reality simulation represent technology that offers a fully immersive experience with the use of a headset. Virtual simulation has been considered as an effective strategy to enhance metacognition[8–10] as well as a rich learning environment by means of a realistic and reflective experience. Unlike the conventional two-dimensional text or image-based abstract e-learning lesson as a learning environment, current virtual simulation comprises various advanced technologies including colorful images through 3D graphics, color and sound processing technology, and—as the most innovative aspect—various interactive opportunities [11]. The learner interacts directly with the target, which can lead to high commitment and participation, as well as immersive learning. In the virtual space where learners act through avatars instead of themselves, remote synchronized cooperative interaction between avatars becomes possible [11]. Interaction using the avatars in virtual simulation could derive high presence of participants[12]. These interactions can stimulate learning participation through similar means as the traditional face-to-face cooperative encounters. The virtual environment with multi-user participation is also beneficial as an educational platform since it enables instant reactions and feedback. It is more flexible and open to access so that objects in the virtual environment can be recycled; and the environment comprising the generated objects is familiar, which makes the learner feel comfortable, and the hardware construction cost is effectively lower in general [13].

Second Life(Linden Lab, San Francisco, CA) and CliniSpace (Innovation in learning, Toronto, Ontario) using Unity are the multi-user virtual simulation platforms widely used as educational tools [14]. Users can create their own avatars and design spaces and tasks that the avatars can use. Users of those platforms also communicate with other users globally, demonstrating the possibility of users having multiple potential experiences in the real world and how it can overcome the temporal and spatial limitations of existing learning methods. Thus, the possibilities of the multi-user virtual environment, such as Second Life and others, were considered as the next-generation education techniques.

Virtual simulations are being used as educational materials and are positively evaluated as through a systematic review [15]. However, the best curriculum model that can be applied in nursing education has not yet been explored and presented. This curriculum requires instructors to set learning objectives for the competencies that learners ultimately need to inculcate to teach effectively and enables learners to identify and cultivate these competences for effective learning. Previous research on nursing simulation suggested a taxonomy for games and simulation as clinical, pathophysiology, and psychosocial domains[16]. Wilford et al.[17] reported simulation curriculum in nursing as including professional and ethical practice, care delivery, care management, and personal and professional development.

This study aimed to develop a clinical curriculum to meet the competencies that could be taught with virtual simulation for undergraduate programs. Specifically, it aimed to develop curriculum content, structure and representative topics for virtual simulation in nursing education, and evaluate the developed curriculum model.

## Methods

### The aim

The aim of study was to develop curriculum content, structure and representative topics for virtual simulation in nursing education, and pilot-evaluate the developed curriculum model.

### Research design

A curriculum development process [18] and a pilot evaluation study was used. The process involves setting the groundwork for change, creative process of visioning, choosing organizing parameters, generating curriculum design, crafting courses, and implementing and evaluating the curriculum.

The first phase is setting the groundwork for change to identify nursing competencies and appropriate competencies in virtual simulation. In this phase of creating the vision and choosing structural parameters, we classified nursing topics and skills appropriate for virtual simulation. Then, we developed the curriculum structure, organized the details of the contents for virtual simulation, and prepared appropriate instructional material and methods to generate curriculum design. In the next stage of crafting, implementing, and evaluating courses, we assessed the validity of the designed curriculum using experts from the field and applied it to the actual curriculum. Final stage of the study included nursing students’ evaluation of the virtual simulation prototypes.

### Participants

Fourteen nurses with working experience for more than five years, eight nursing faculty members, simulation experts who had simulation education experience for more than five years, and three information experts participated in the focus group interviews. Thirty-five senior nursing undergraduate students in an university participated in the evaluation of the developed prototypes of the curriculum. There were 30 female and five male students and their average age was 22.9 years old.

## Data Collection

### Literature review

Previous studies applying virtual simulation were reviewed along with potential topics for virtual simulation from the major nursing classification systems.

**Fig. 1:**
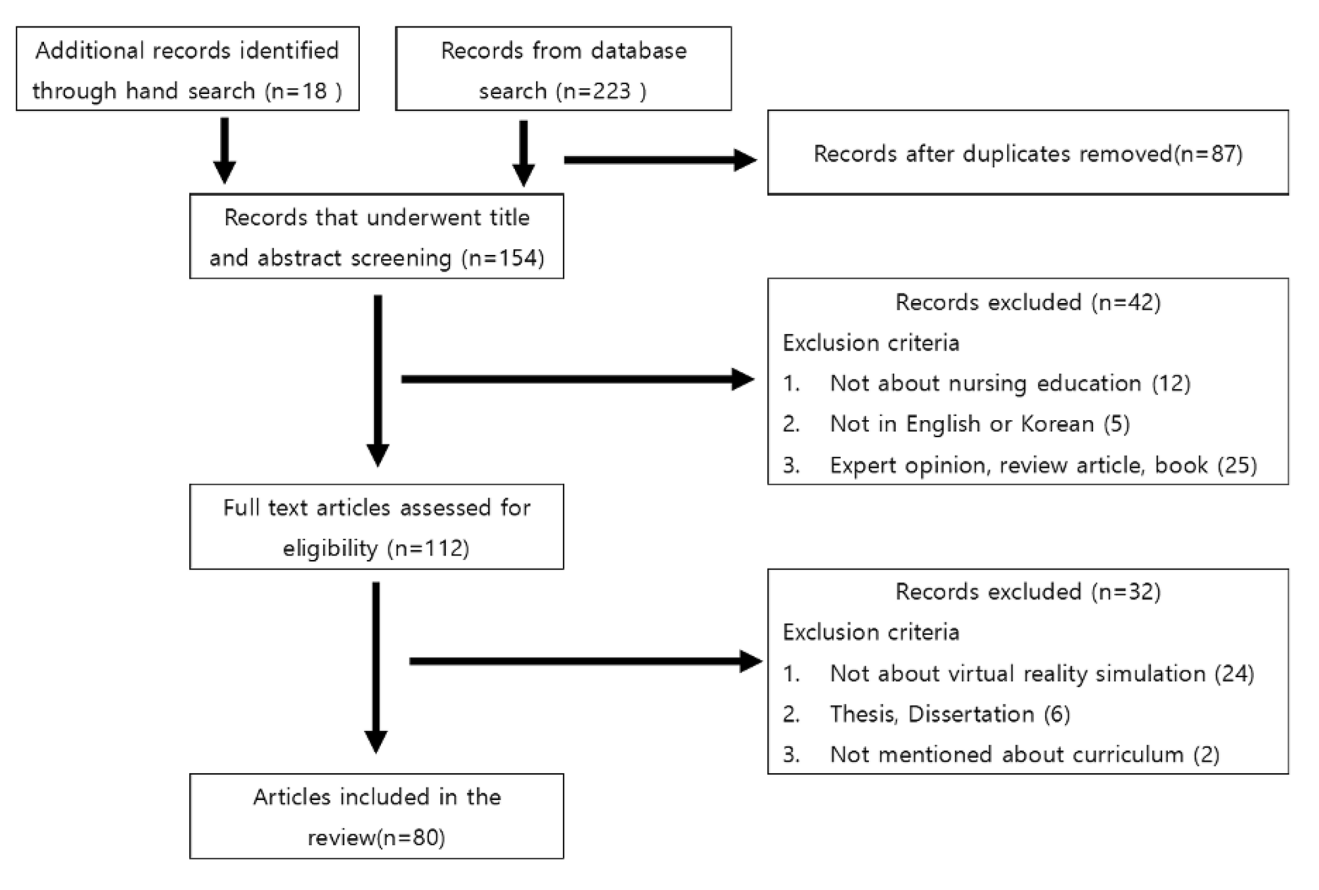
Literature review flow

The inclusion criteria for the literature review were: (1) papers published in academic journals from January 1, 2010 to December 30, 2017; and (2) research in nursing or a nursing environment. The exclusion criteria were (1) reviews, expert opinions, and books; (2) unpublished theses; (3) no mention of the curriculum, and (4) research using virtual simulation not matching the definition of virtual simulation used in this study. The search string for PubMed and Medline were as follows: ((“virtual” (Title/Abstract)) AND (“nursing”(Title/Abstract)) AND ((“patient simulation”[MESH]) OR (“simulation training”[MESH])) OR (“computer simulation”[MESH])). The search string for CINAHL was: ((“virtual”(Title/Abstract)) AND (“nursing”(Title/Abstract)) AND (“patient simulation”[CINAHL HEADINGS]) OR (“computer simulation”[CINAHL HEADINGS])). There was a total of 241 articles initially identified, and 80 were used for analysis according to the systematic extraction process (Fig. 1).

In the nursing taxonomy, topics that can be applied to virtual simulation were extracted based on International Classification for Nursing Practice (ICNP), NANDA, Gordon’ s Functional Health Patterns, and the integrated nursing job analysis framework presented by the Korean National Health Examiner. Based on the characteristics and advantages of virtual simulation, we extracted key themes. Characteristics and merits of virtual simulation such as reproducibility, accessibility, safety, repeatability, and cost-effectiveness were considered when analyzing the core topics in the virtual simulation curriculum.

### Focus Group interviews

To derive the virtual simulation topics, participants were interviewed using a semi-structured questionnaire on the appropriate subject for virtual simulation[19]. A brainstorming workshop and three focus group interviews were held from January to April 2017. In the two-hour brainstorming workshop, participants were asked to answer and discuss the adequate topics or subjects for nursing education using virtual simulation and then list major nursing topics or skills that can be developed through virtual simulation. Three focus group interviews were conducted face to face and lasted 40–60 min. The questions for focus group interviews were generated from the brainstorming workshop and they were: “What do you think is the most important topic in nursing education using virtual technology?”, “What do you think is a topic that can be implemented as a virtual technology?,” and “If you have preceptor experience, what should preceptees learn repeatedly?.”

### Crafting, implementing, and evaluating courses of the virtual simulation curriculum

The generative process of curriculum design included setting learning outcomes at the program level, defining concepts or content for mapping, identifying delimiting factors, and creating an overview of courses. To develop the curriculum model, components of educational strategies were included as a framework containing domains, subdomains, and representative topics for virtual simulation.

We developed courses using representative topics and teaching and learning strategies in the developed curriculum and evaluated the developed scenarios. Then, nine prototypes of scenarios in the developed curriculum were constructed. The representative scenarios for each domain of the developed curriculum were written under the guidance of the developed templates for virtual simulation[20] and 3D modeling. We also conducted content validity by nine simulation experts with more than five-year experience in nursing education to evaluate the developed curriculum model as well as nine prototypes.

**Fig. 2:**
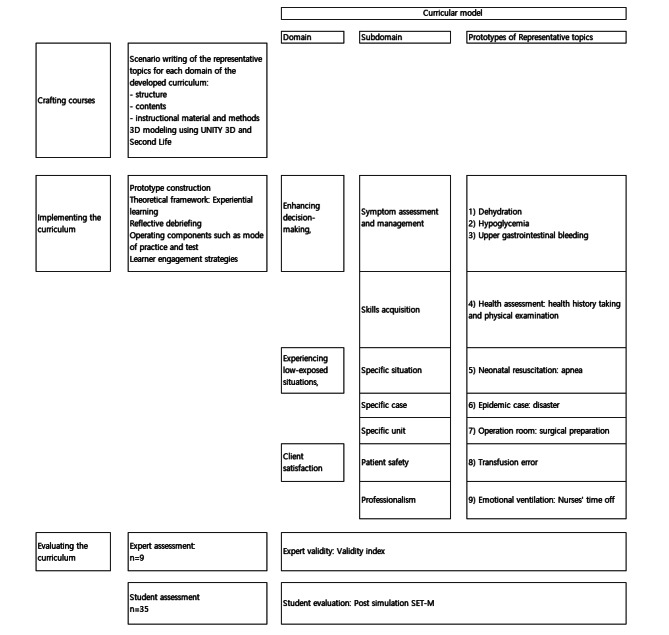
Study flow of implementing and evaluating the curricular model

35 nursing undergraduate students were recruited from an university in Seoul to evaluate the prototypes of virtual simulation in the developed curricular model. After using the prototypes, students evaluated the effectiveness of simulation using the modified version of Simulation Effectiveness Tool developed by Leighton et al. (2015) [20]. This tool consists of 19 items and each item was measured on a three-point scale from “Do not agree” (1 point) to “somewhat agree” (3 points). The Cronbach’s α for 19 items was 0.943 and the Cronbach’s α for four sub-factors were 0.833 in pre-briefing for 2 items, 0.852 in learning (6 items), 0.913 in confidence (6 items), and 0.908 in debriefing (5 items). Figure 2 shows the study flow of implementing and evaluating the curricular model.

### Data analysis

The data from focus group interviews and workshops were analyzed using content analysis to identify topics of virtual simulation. In this study, content analysis included active reading, verification, correction, modification, and organization of data. After transcribing the audio-recorded interviews, a sense of the whole was obtained by reading the transcripts several times. The unit of analysis was the word, and meaningful words and phrases from the interviews were categorized [21]. The data analysis was conducted manually without a computer software.

All three researchers were familiar with the virtual simulation and had previous experiences in content analysis research. The researchers independently reviewed the interview transcripts and collected similar meaningful words from sentences and coded them as one representative word. The researchers’ coding continued until inter-rater reliability was greater than 90%. As the content analysis proceeded, the collected data were categorized based on relevance and similarities. Researchers repeatedly reviewed, discussed, and agreed upon the major domains, subdomains, and representative topics.

### Methodological rigor

To achieve methodological rigor regarding the qualitative data, only the topic elements and meaningful phrases were included in the results[22]. Concerning trustworthiness, credibility was achieved due to the extensive research experience in nursing education and virtual simulation of the study team. Transferability was achieved through adequate selection of participants and experts, data collection procedures, and data analysis processes. Moreover, confirmability was accomplished through the two coders’ repeated review of the descriptive data to verify the coding.

## Results

### Topics for virtual simulation from literature review

Topics in literature were retained after a relevancy screening and then categorized based on the content areas of disease, symptom, psychosocial issues, communication, community health care, skill, customer satisfaction, knowledge, and critical care from previous studies and symptoms, specific cases, routine tasks, and patient safety from major nursing classification systems.

### Topics for virtual Simulation in focus group interviews

Focus group participants reported a wide range of topics as major contents for virtual simulation in nursing education. They are nursing care in specific situations, experiencing the routine tasks of nurses, mastering nursing skills, symptom management, assuring patient safety, knowledge acquisition, using intensive equipment, and giving emotional care. [Supplementary file 2 here]

### Curriculum content for virtual simulation

Three core domains of curriculum content for virtual simulation (Table 1) derived from major topics were included: enhancing decision-making, experiencing low-exposed situations, and building professional resilience. The domain of enhancing decision-making contains two subdomains: symptom assessment and management, and skills acquisition. Representative topics for symptom assessment and management included management of fever, bleeding, and ineffective respiration. Using intensive medical equipment such as the ventilator and extracorporeal membrane oxygenation as well as health assessment represented the subdomain of skills acquisition.

**Table 1:** Curriculum content and structure of virtual simulation in nursing

Domains	Subdomains	Representative topics	
Enhancing decision-making	Symptom assessment and management	Fever Bleeding Ineffective respiration Constipation Urinary incontinence Shock Dizziness	Diarrhea Neonatal jaundice Ineffective tissue perfusion Unstable blood glucose Imbalanced fluid volume
	Skill acquisition	Equipment management Ventilator ECG^2^ CRRT3	ECMO^1^ Health assessment Fundamental nursing
Experiencing low-exposed situations	Specific situation	Unpredictable situation management Patient safety Cardiopulmonary resuscitation	Terminal care Vulnerable community outreach
	Specific case	Parenting Mental disorder AIDS^4^	
	Specific unit	Operation room Emergency room (Triage)	
Building professional resilience	Patient safety	Bed sore Transfusion Fall	
	Quick stress relief	Emotional ventilation Nurses’ time off Self-soothing strategy	Information sharing Time management

^1^ Extracorporeal membrane oxygenation, ECMO; ^2^Electrocardiogramm, ECG; ^3^Continuous Renal Replacement Therapy); ^4^Acquired Immune Deficiency Syndrome, AIDS

### Evaluation of the Curriculum Content and structure: content evaluation by Expert Panels

Table 2 shows the scores of content validity of the developed topics including major domains, subdomains, and representative topics for the virtual simulation curriculum in nursing education. The scale’s content validity index/universal agreement (S-CVI/UA) calculation method was used to check S-CVI of the developed curriculum components. A score of 0.80 or greater is recommended as a percentage of all 3 or 4 questions by all experts[19], confirming that all components had values greater than 0.80. Descriptive feedback from expert panels on the developed curriculum was analyzed.

**Table 2:** Expert validity of the developed contents and structure of the virtual simulation using multiuser virtual environment curriculum (n = 9)

Domain Subdomain		Acceptance i-CVI^1^	Importance i-CVI	Accordance i-CVI
Classification of taxonomy	Enhancing decision-making Experiencing rare situation Building professional resilience	0.89	0.89	1
Enhancing decision-making	Symptom management	1	1	1
	Skill acquisition	1	1	1
Experiencing rare situation	Specific situation	1	1	1
	Specific case	1	1	0.89
	Specific unit	1	1	1
Building professional resilience	Patient safety	1	1	1
	Quick stress relief	1	1	0.89

^1^ item-level content validity index

### Creating, implementing, and evaluating courses of representative topics

We selected nine representative topics from the curriculum contents for virtual simulation. The nine representative topics include dehydration, hypoglycemia, and upper gastrointestinal bleeding scenarios (symptom management under enhancing decision-making); health assessment scenarios (skills acquisition under enhancing decision-making); apneotic event management and epidemic disaster scenarios (experiencing low-exposed situations); experiencing the operating room scenario (experiencing low-exposed situation); emotional ventilation, otherwise known as the nurses’ time off, scenario (quick stress relief under building professional resilience); and a scenario of detecting and reporting errors in transfusion (patient safety under building professional resilience).

**Fig. 3:**
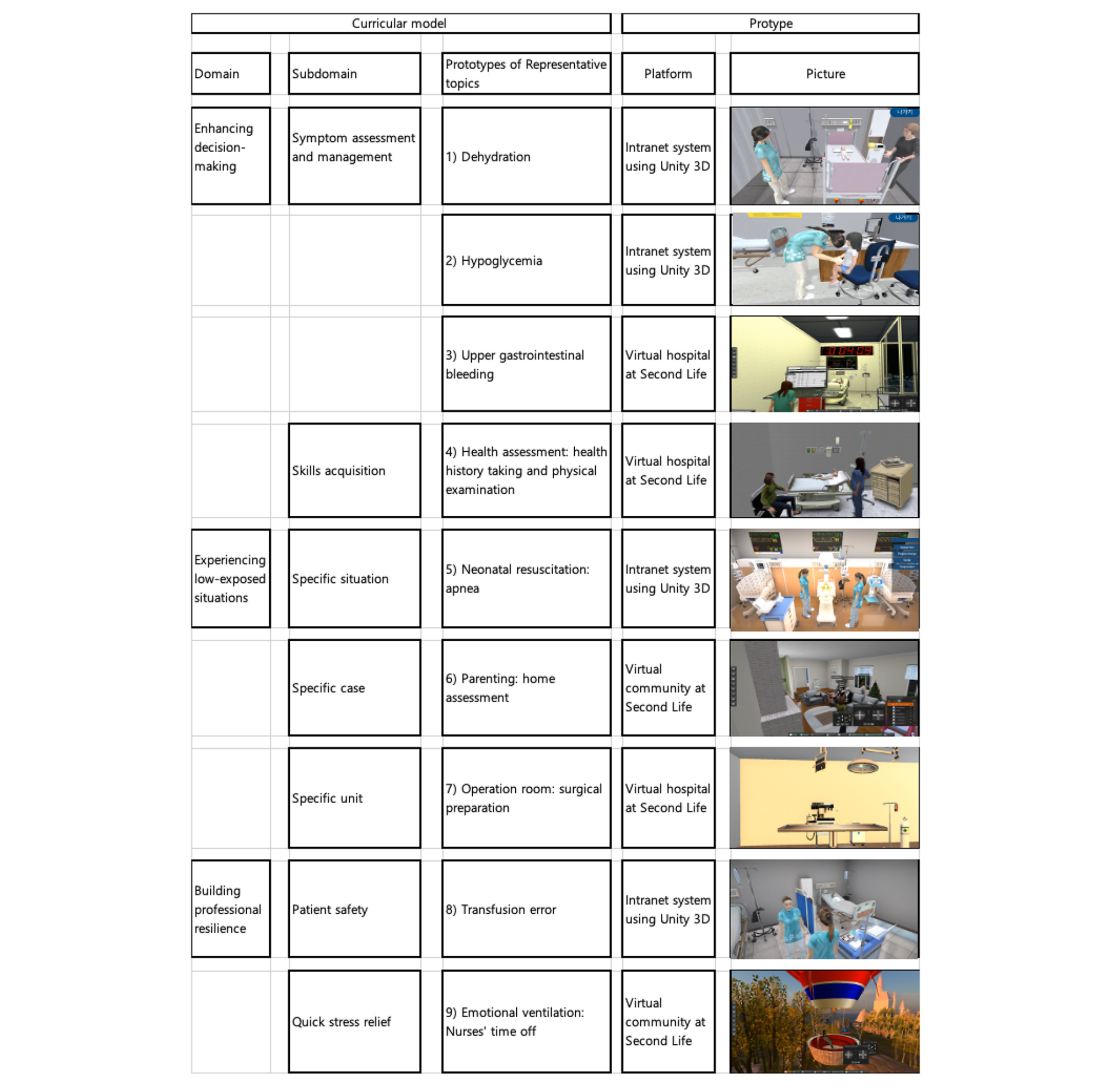
Represented prototypes of developed scenarios

We built two virtual environments and constructed nine scenario prototypes (Fig. 3). They were five prototypes in a virtual community at Second Life and four prototypes in a virtual program platform constructed with Unity 3D. The virtual environment in Second Life comprised a three-story acute care hospital, remote village having a small health post, disaster area, and nurses’ resting area. The virtual hospital involved three layers in total. The first floor contained the orientation information such as the proper keyboard operation and the item operation method in virtual space. The second floor consisted of a nurse station, five rooms, and a debriefing room. Each room had its own scenario environment. On the third floor of the virtual hospital, the large display boards were linked to Internet resources for the pre-learning for each scenario. The information was classified and displayed according to each scenario so that learners could view the linked materials by expanding them when they clicked. In addition, the skill lab was organized in separate classrooms in order to operate and practice the items necessary for each scenario and provide spaces for adaptation to the scenario progression.

In a virtual program built using Unity 3D, there were four scenarios to experience in the virtual hospital. Each simulated scenario consisted of a practice and test mode, and learners could take two practices and one test for each scenario. In practice mode, learners could take time to adapt to the progress of the simulation by operating the various objects in the virtual environment. In the test mode, virtual simulations could only be operated for a set time, and other colleagues and professors could provide feedback on the learners’ nursing behavior while experiencing the simulations. Students who completed the simulation in test mode could check the feedback by other colleagues or professors after self-reflection. After self-reflection and checking of the feedback, learners and professors were expected to gather in the debriefing room to reflect on the learning process and share their experiences and opinions.

### Operating components and learner engagement strategies

In the present virtual simulation prototypes, we developed the program using both engines of Second Life and the intranet system using UNITY 3D. Unity 3D was used for potential expansion toward virtual reality simulation, but the current program was built and operated in the form of screen-based partially immersive simulation due to its learner accessibility and system stability. Action strategies for effective education included face-to-face meetings, operation, concept mapping, recording the performance, self-evaluation and reflection, and experiential learning. Overall, scenarios applied regular mode of representation and interaction using avatars and two-way interaction.

In the virtual campus, students enter the program using their ID and password. They then choose their own avatar and assigned scenario. After having the short tutorial session on navigating in the virtual world, students could go to the pre-briefing room to check related guidelines and protocols. Afterwards, they could then go to the patient situations alone or in groups to solve a patient case. There were two modes to experience the scenario for students, one for practicing multiple times and one for testing. Students could experience both modes. After the appropriate nursing interventions, they then self-reflect using the Situation-Background-Assessment-Recommendation format and concept mapping before they have the group-debriefing session.

In this developed simulation curriculum, the learning outcomes included enhancing metacognitive abilities of nursing students. To achieve the desired learning outcomes, all virtual simulation scenarios and prototypes were constructed using the framework of experiential learning. Participants were expected to have concrete experience in virtual environments, and then through reflective debriefing, guided-structured self-reflection, and group reflection, they had opportunities to engage in abstract conceptualization. The debriefing included reflective observation using feedback and guidance with concept mapping and clinical judgment rubric for recorded performance. In the subsequent virtual simulation experience in other scenarios, they had more opportunities for active experiments in further simulations rather than real life using their established schema. This circle of experiential learning as designed to promote learners’ metacognitive competence to augment the clinical experience.

Evaluation of the developed nine prototypes by expert panels and students.

**Fig. 4:**
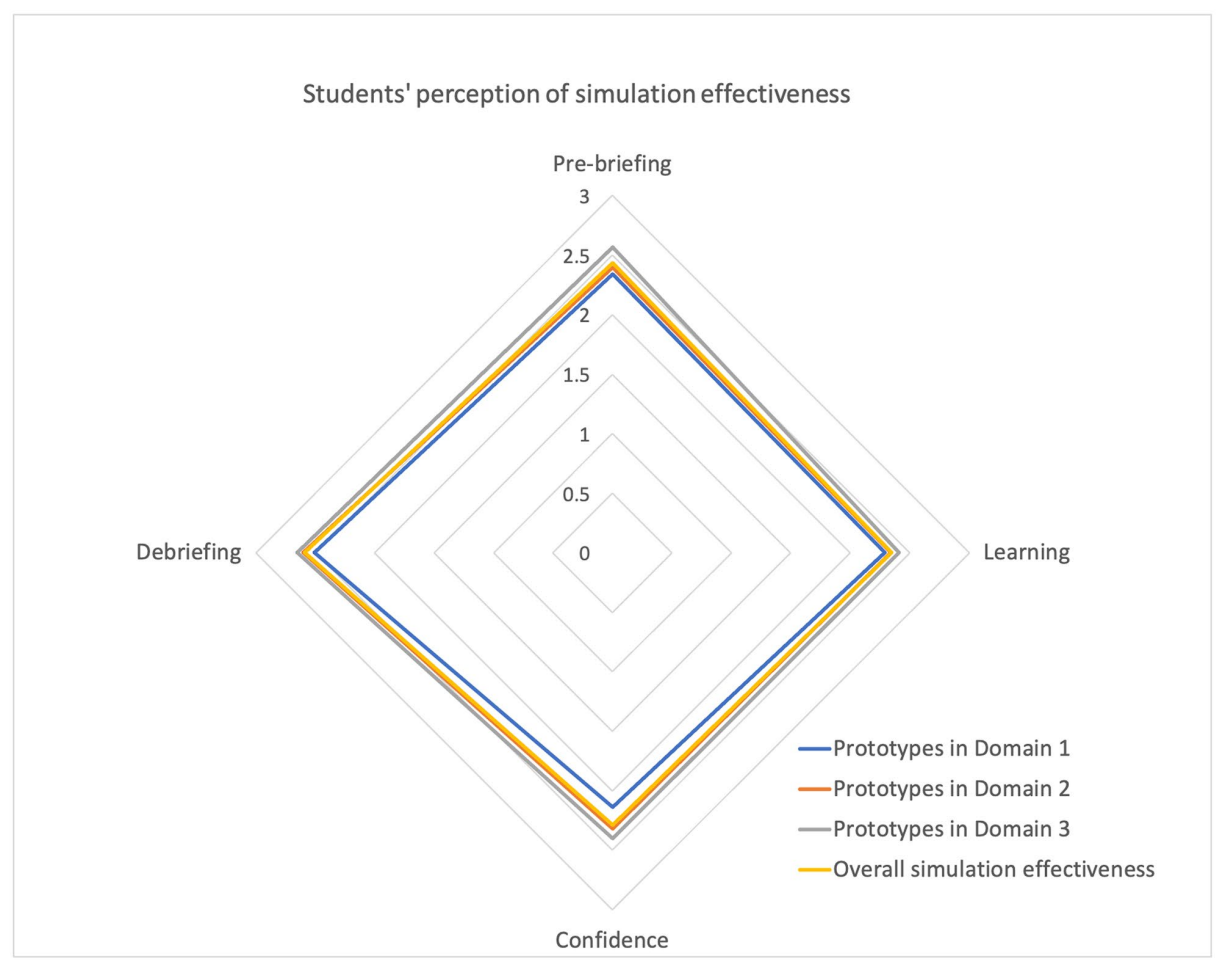
Students’ perception of simulation effectiveness

The validity of the contents of the developed nine prototypes was tested through consultation with nine expert panels. The content was reviewed for items that fell under 1.00 [19] when it was judged by expert panels (Supplementary file 3). Students used the representative prototypes of virtual simulation and reported the simulation effectiveness. The first domain was clinical decision making, the second was low exposed situations, and the third was building professional resilience. There were no low responses under two in score of each subfactor. Figure 4 shows students’ reported simulation effectiveness after using the prototypes (Fig. 4).

## Discussion

Virtual simulation in nursing education was developed in the recent decade, so the content areas and educational strategies using information and communication technology (ICT) are still preliminary in the academic context including technological disciplines [23, 24]. Constructing a curriculum model for virtual simulation can facilitate the development of sound educational courses in virtual simulation. Considering the current need to develop more enabling and participatory strategies for nursing education and the development of well-designed virtual simulation has resulted in positive learning effects [7, 25], it is necessary to construct and apply an adequate curricular model for virtual simulation in nursing education.

Curriculum design models such as blocked, theory-based, concept-based, and competency-based curricula have been used in nursing faculties for designing undergraduate curricula[26]. To focus on the learners’ acquisition of desired measurable competencies leading to expected graduate outcomes, competency-based curricular models are introduced and used in academic progression models in nursing[26]. A recent study [27] reported that competency-based nursing education was the current need in facilitating nursing care and practice. According to Lau et al.[27], major competencies for the nursing curriculum include communication, knowledge application, health assessment, nursing process, critical thinking, reflective practice, documentation, and interpersonal skills. In their following study, they found that the core curricular topics should include patient engagement, patient care and practice, care management, common procedures, safety, urgent care, transition care, patient education, interprofessional collaboration, and palliative care [27].

Compared to the previously addressed competencies in nursing education, the structural components (i.e., clinical decision-making using symptom management and skill acquisition, experiencing low-exposed situations, building professional s in nursing education, and patient safety such as error identification and reporting) for virtual simulation generated in this study are consistent with the components presented by previous nursing curriculum models [27], specifically, metacognitive competencies and learner engagement. Medina, Castleberry, and Persky[2] identified that current medical errors were related to health care providers’ cognitive issues and metacognitive monitoring and evaluation could reduce those medical errors effectively [2].

Currey, Massey, Allen, and Jones[28] stated that introducing curricula focused on educating nurses on the specific requirements of assessing, managing, and evaluating is important. This is one curriculum focusing on the specific educational purpose of enhancing metacognitive abilities of nursing students. Additionally, newly reported nursing curriculum mostly focused on the nursing graduates’ performance competence. Students participated the developed virtual simulation prototype expressed their immersive experience. As they showed high satisfaction with learning and confidence, the developed curriculum model might be learning process to develop nursing competencies for learners. Considering that the main expectation for virtual simulation is to capture students’ performance through active engagement and participation in the learning experience, the developed nursing curriculum is expected to serve as a supplementary method for current nursing education. The scenarios in the developed curriculum may focus on core competencies in nursing practice and enable nurse educators to introduce these core competencies in their education.

To perform effective virtual simulation, presence, affordance, and immersion were identified as critical components in virtual simulation in a previous research. The learners participation, engagement, and experience results in the achievement of learning outcomes [14]. In this regard, learner engagement was one of the main operating components in the developed curriculum. The final learning outcome of virtual simulation curriculum can be accomplished by means of designed operating components and learner engagement strategies. In the present study, nine representative topics in the new curriculum were presented and constructed in the Second Life and the Unity 3D program platforms. Through these prototypes of virtual simulation, we can identify the basic mechanisms and design characteristics of the curriculum components, as well as validate the curriculum. Although the current level of virtual simulation modeling has several limitations including deficient use of ICT, artificial intelligence, well-developed templates[20], and trained instructors[29], virtual simulation can help nurse educators design more effective ways of enhancing metacognitive abilities by increasing learner engagement.

### Strengths and Limitations

As the current nursing educators may be unfamiliar with virtual environments, application of the developed curriculum might not be practical. In particular, many faculty members felt challenges to apply virtual simulation to education in a recently limited environment[30]. Actualization of the nine prototypes of virtual simulation scenarios can help future researchers and educators to design and plan its adaptation and utilization in the nursing education.

This study has a few limitations. The curriculum content may not be applicable in other nursing jurisdictions. In addition, although there was no major problem in the overall external environment configuration, the virtual items limited the implementation of the scenarios and nursing activities in detail, and it was difficult to learn effectively due to the screen configuration and item manipulation method. Educators need to employ sustainable strategies when using this type of simulation. Further studies are warranted to define the outcomes of the developed curriculum and to assess the enabling environment for virtual simulation in nursing, including appropriate server capacities, devices, and coding abilities. Finally, the absence of any measures of knowledge, skill acquisition and/or student preparedness for practice is another limitation of this study although students’ evaluation of prototypes was performed. Future studies on the effect of these curricular based virtual simulation needs to introduce more comprehensive assessment according the Kirkpatrick hierarchy and to explore these curricular results.

## Conclusions

Virtual simulation has been emphasized by pandemic disaster when clinical practicums could not be provided in clinical and community institutions. Considering how to move forward with appropriate content for nursing programs with current form of educational strategies of mannequin-based simulation, virtual-based simulation, and/or hands-on clinical experiences, there is an urgent need to examine the use of virtual simulations and their role in a nursing curriculum given the pandemic and how nursing programs have had to move to online curricular/simulation experiences.

In the study, we suggest that virtual simulation can provide nursing education with empowered educational strategies for enhancing metacognition as a core nursing competence. Considering that education in nursing and other health care professions is encountering new demands and challenges from learners and the changing society, new approaches and different curriculum can help nurse educators to improve educational outcomes for their students.

## Electronic supplementary material

Below is the link to the electronic supplementary material.

Supplementary Material 1

## Data Availability

All data set and materials are available from the corresponding author.
