# Supplementary material for: Development and assessment of a curriculum model for virtual simulation in nursing: curriculum development and pilot-evaluation
Source: BMC Med Educ. 2023 Apr 26;23:284. doi: 10.1186/s12909-023-04283-4 (PMC10134613; doi:10.1186/s12909-023-04283-4)
Supplement: Supplementary file 1 — Supplementary Material 1 [file 12909_2023_4283_MOESM1_ESM.docx]

Supplementary file 1 Topics from literature on virtual simulation

| **Resource** | **Topics** | | **Categories** |
| --- | --- | --- | --- |
| Topics from previous studies | Prostate cancer  Diabetes  Rheumatoid arthritis  Bronchiolitis  Cystic fibrosis  Pneumonia  Asthma  Cystic fibrosis  Respiratory distress syndrome  Wet lungs  Heart failure | Sepsis  Cardiopulmonary arrest  Dementia  Preeclampsia  Guillain-Barre Syndrome  Suspected gluten intolerance  Obesity  Growth hormone deficiency  Acute coronary syndrome  Hypoglycemia  Pyelonephritis | Disease |
|  | Abdominal pain  Continence management and urinary concerns  Hyperbilirubinemia  Low blood glucose  Anaphylaxis condition  Chest discomfort  Dyspnea | Learning disabilities  Airway obstruction  Breathlessness  Hypotension  Tachycardia  Oliguria  Altered consciousness  Abnormal temperatures | Symptom |
|  | Long term effects and end of life care | Psychosocial issues | Psychosocial issues |
|  | Journal club  Interprofessionalism  Health assessment interview skill  Communication with elderly patient | Mental health nursing, counseling  Discussion  Interpersonal relationship  Conflict management | Communication |
|  | Domestic violence  Tot | Disaster training | Community  Healthcare |
|  | The disaster-specific skill of decontamination | Health assessment skill  Decontamination | Skill |
|  | New facility startup  Experience of the virtual world | Emotional engagement  Patient safety  Person-centered care | Customer satisfaction |
|  | Biphosphonate for osteoporosis | 5-alpha reductase inhibitor for prostatic hyperplasia | Knowledge |
|  | Intermediate life support |  | Critical care |
| Topics from major nursing classification system | Ineffective airway clearance  Impaired gas exchange  Ineffective tissue perfusion (brain, peripheral, renal, cardiovascular, pulmonary)  Constipation  Risk for unstable blood glucose | Diarrhea  Risk for imbalanced body temperature  Impaired swallowing  Ineffective breathing pattern  Impaired urinary elimination  Imbalanced fluid volume  Acute confusion | Symptom |
|  | Cardiopulmonary resuscitation  Community nursing  Disaster nursing | Nursing care of children with HIV and AIDS  Prenatal nursing care | Specific case |
|  | Handover  Cooperation (team work) | Nursing skill  Medication | Routine task |
|  | Transfusion | Fall | Patient safety |

Supplementary file 2 Topics for virtual simulation in nursing education derived from focus group interviews and workshops

| **Topics** | | **Categories** |
| --- | --- | --- |
| Emergency room (triage)  Cardiopulmonary resuscitation  Intensive care unit | Operation room  Disaster | Nursing care in specific situations |
| Handover  Nursing Record | Assessment  Priority | Experiencing nurse’s routine task |
| Suction  Foley  Enema | Medication  Intravenous  Medication | Mastering nursing skills |
| Bleeding  Shock  Low/High blood pressure  Fever  Sepsis  Dyspnea  Dysuria | Desaturation  Chest pain  Abdomen pain (appendicitis, colitis, acute pyelonephritis, bowel perforation, medication side effect)  Mental disorders | Symptom |
| Infection  Medication error | Infection management  Nursing Negligence (Medical malpractice) | Assuring patient safety |
| Pharmacology  Pathophysiology | Interpretation of examinations (radiology, labs, etc.) | Knowledge acquisition |
| Ventilator  EKG | CRRT  ECMO | Using essential equipment |
| Time management  Getting help after nursing malpractice | Emotional ventilation from death  Emotional ventilation from nurse burnout | Giving emotional care |

Supplementary file 3 Expert validity of the curriculum outline of representative scenarios

(n=9)

| **Domain** | **Subdomain** | | **Scenario topic** | **Outlines** | **Acceptance**  **i-CVI** | **Importance**  **i-CVI** | **Accordance**  **i-CVI** |
| --- | --- | --- | --- | --- | --- | --- | --- |
| Enhancing decision-making | Symptom management | Imbalanced Fluid Volume | Dehydration | Learning objectives | 0.89 | 1 | 1 |
|  |  |  |  | Course flow | 1 | 1 | 1 |
|  |  |  |  | Evaluation | 1 | 1 | 1 |
|  |  |  |  | Representation and interaction | 1 | 1 | 1 |
|  |  | Unstable blood glucose | Hypoglycemia | Learning objectives | 0.89 | 1 | 1 |
|  |  |  |  | Course flow | 1 | 1 | 1 |
|  |  |  |  | Evaluation | 1 | 1 | 1 |
|  |  |  |  | Representation and interaction | 1 | 1 | 1 |
|  |  | Bleeding | Upper gastrointestinal bleeding | Learning objectives | 0.89 | 1 | 0.89 |
|  |  |  |  | Course flow | 1 | 1 | 1 |
|  |  |  |  | Evaluation | 1 | 1 | 1 |
|  |  |  |  | Representation and interaction | 1 | 1 | 1 |
|  | Skill acquisition | Health assessment | Health history and physical exam | Learning objectives | 1 | 1 | 1 |
|  |  |  |  | Course flow | 1 | 1 | 1 |
|  |  |  |  | Evaluation | 1 | 1 | 1 |
|  |  |  |  | Representation and interaction | 1 | 1 | 1 |
| Experiencing rare situation | Specific situation | Cardiopulmonary resuscitation | Apnea | Learning objectives | 1 | 1 | 1 |
|  |  |  |  | Course flow | 1 | 1 | 1 |
|  |  |  |  | Evaluation | 1 | 1 | 1 |
|  |  |  |  | Representation and interaction | 1 | 1 | 1 |
|  |  | Disaster | Epidemic | Learning objectives | 1 | 1 | 1 |
|  |  |  |  | Course flow | 1 | 1 | 1 |
|  |  |  |  | Evaluation | 1 | 1 | 1 |
|  |  |  |  | Representation and interaction | 1 | 0.89 | 1 |
|  | Specific unit | Operating room | Surgical preparation | Learning objectives | 0.89 | 1 | 0.89 |
|  |  |  |  | Course flow | 1 | 1 | 0.89 |
|  |  |  |  | Evaluation | 0.89 | 0.89 | 0.78 |
|  |  |  |  | Representation and interaction | 1 | 1 | 0.89 |
| Client satisfaction | Professionalism | Emotional ventilation | Nurses’ time off | Learning objectives | 1 | 1 | 1 |
|  |  |  |  | Course flow | 1 | 1 | 1 |
|  |  |  |  | Evaluation | 1 | 1 | 1 |
|  |  |  |  | Representation and interaction | 1 | 1 | 1 |
|  | Patient safety |  | Transfusion | Learning objectives | 0.89 | 1 | 1 |
|  |  |  |  | Course flow | 1 | 1 | 1 |
|  |  |  |  | Evaluation | 1 | 1 | 1 |
|  |  |  |  | Representation and interaction | 1 | 1 | 1 |
